# ZNF423: A New Player in Estrogen Receptor-Positive Breast Cancer

**DOI:** 10.3389/fendo.2018.00255

**Published:** 2018-05-18

**Authors:** Heather M. Bond, Stefania Scicchitano, Emanuela Chiarella, Nicola Amodio, Valeria Lucchino, Annamaria Aloisio, Ylenia Montalcini, Maria Mesuraca, Giovanni Morrone

**Affiliations:** ^1^Laboratory of Molecular Haematopoiesis and Stem Cell Biology, Department of Experimental and Clinical Medicine, Magna Græcia University of Catanzaro, Catanzaro, Italy; ^2^Laboratory of Medical Oncology, Department of Experimental and Clinical Medicine, Magna Græcia University of Catanzaro, Catanzaro, Italy

**Keywords:** breast cancer, cathepsin O, calmodulin like 3, estrogen receptors, single nucleotide polymorphisms, selective estrogen receptor modulators, ZNF423, ZNF521

## Abstract

Preventive therapy can target hormone-responsive breast cancer (BC) by treatment with selective estrogen receptor modulators (SERMs) and reduce the incidence of BC. Genome-wide association studies have identified single nucleotide polymorphisms (SNPs) with relevant predictive values, SNPs in the *ZNF423* gene were associated with decreased risk of BC during SERM therapy, and SNPs in the *Cathepsin O* gene with an increased risk. ZNF423, which was not previously associated with BC is a multifunctional transcription factor known to have a role in development, neurogenesis, and adipogenesis and is implicated in other types of cancer. ZNF423 is transcriptionally controlled by the homolog ZNF521, early B cell factor transcription factor, epigenetic silencing of the promoter by CpG island hyper-methylation, and also by ZNF423 itself in an auto-regulatory loop. In BC cells, ZNF423 expression is found to be induced by estrogen, dependent on the binding of the estrogen receptor and calmodulin-like 3 to SNPs in *ZNP423* intronic sites in proximity to consensus estrogen response elements. ZNF423 has also been shown to play a mechanistic role by trans-activating the tumor suppressor BRCA1 and thus modulating the DNA damage response. Even though recent extensive trial studies did not classify these SNPs with the highest predictive values, for inclusion in polygenic SNP analysis, the mechanism unveiled in these studies has introduced ZNF423 as a factor important in the control of the estrogen response. Here, we aim at providing an overview of ZNF423 expression and functional role in human malignancies, with a specific focus on its implication in hormone-responsive BC.

## Introduction

Breast cancer (BC) is the malignancy with the highest incidence among women and even though there have been improvements in diagnosis and treatment, it is still the leading cause of cancer deaths in women, indeed, it accounts for 7% of all cancer deaths. In the USA from 2008 to 2012, BC represented 20% of the cancers diagnosed (1), and it is estimated that, in 2018, there are 266,120 new cases of BC and 40,920 deaths in the USA (2). In many cases of estrogen positive (ER+) BC, the E_2_–estrogen receptor (ER) complex is involved in malignant transformation and progression, and can be therapeutically targeted with selective estrogen receptor modulators (SERMs) such as tamoxifen, which has been used for the last three decades. This treatment has been approved by the US FDA also for treatment to be used in a preventive context, where women at high risk are treated with SERMs to reduce the probability of developing BC. There have been several prospective randomized clinical trials to test the efficacy of the SERM tamoxifen (3–5), which gave varying degrees of prevention with a decreased incidence of up to 38%. However, there have also emerged concerns for toxicities including cases of endometrial cancer, venous thromboembolism, and menopausal symptoms, which were observed in these trails (3). To improve risk-benefit, several phase II trials have already demonstrated that low-dose tamoxifen retains biological activity while potentially resulting in lower toxicity (4). The benefits, safety, and cost-effectiveness should be improved if the population treated are stratified such that preferentially patients only with the appropriate high risk are treated. Risk factors, including age, family history of breast or ovarian cancer, menopausal status, body mass index, use of hormone replacement, age of first child birth, and prior proliferative benign breast disease (6) are combined with mammographic density and genetic mutations. Additionally, prognostic predictive molecular data based on the patients individual genomic profiles where a polygenic analysis of over 100 different single nucleotide polymorphisms (SNPs), which are associated to prediction are being validated (7–10). A combination of these factors, which are statistically analyzed as risk prediction models (11) will facilitate the identification and stratification of patients most likely to benefit from SERM therapy.

It has been found (12) in the *ZNF423* gene that several SNPs in intronic sequences have a predicted decreased risk for BC development, whereas SNPs in the *Cathepsin O* (*CTSO*) gene had an increased risk value. *ZNF423* had not previously been studied in BCs, and since then, several studies, which are discussed, have addressed the mechanisms underlining the SNPs for both *ZNF423* and *CTSO* and point to a relevant role in the response to SERMs (12–15).

*ZNF423* was originally cloned and characterized for its interaction with [Early B cell Factor (EBF)], and then nominated OAZ/ROAZ (rat Olf/EBF-associated zinc finger protein) for its inhibitory activity on olfactory gene expression (16). ZNF423 is a large protein comprising 30 C_2_H_2_ krüppel like zinc fingers (ZNFs) clustering in distinct domains. It has been shown that ZNFs 2–8 are responsible for direct DNA binding to repeated GCACCCn consensus sequences (17), and ZNFs 9–13 are required for the recognition of the bone morphogenic protein (BMP) responsive element, while the interaction with phosphorylated small mother against decapentaplegic (SMAD) proteins involves ZNFs 14–19. The interaction of ZNF423/OAZ with EBF instead requires the C-terminal ZNFs 27–30 (18). In the β-ZNF423 transcript, a 12 amino acid nucleosome remodeling domain (NuRD) is located at the N-terminus (19, 20) and when present, the NuRD sequence derives from an alternative β promoter (21).

ZNF423 has a high degree of homology (65%) with the Zinc finger protein ZNF521, especially within individual ZNFs (22–24). ZNF521 also has a functional NuRD sequence at the N-terminal (25, 26), and this feature is shared by a small family of transcription factors including Friend of GATA1, spalt-like transcription factors 1,2,3 (Sall1,2,3), and B-cell CLL/lymphoma 11A (BCL11/Evi9), known to interact directly with retinoblastoma-binding protein 7/4, a component of the NuRD complex (27). Some functional aspects of ZNF423 and ZNF521 are apparently overlapping, including: the EBF binding and transcriptional inhibition (17, 22, 25, 28) and interaction with SMAD proteins (18, 22); however, ZNF521 is unable to interact with the same DNA consensus recognized by ZNF423 (17). It has been proposed that in addition to forming homodimers, both ZNF423 and ZNF521 can form heterodimers (20). Although some common features, the expression profiles of these proteins, as well as their influence on cancer and differentiation pathways, appear considerably divergent (19, 21, 29).

## ZNF423 in Development and Differentiation

ZNF423 has been shown to play a key role in development and disease (19, 21). On the basis of functional activities so far identified in different cellular systems, it is likely that a definitive picture of the molecular functions of ZNF423 have yet to emerge. A summary of the different activators, inhibitors, and co-interacting transcription factors is shown in Table 1.

**Table 1:** Regulation and activity of ZNF423/zfp423.

	Factor	Pathway	Reference
Activators	BMP2,4	SMAD1,4	(18)
	Retinoic acid	RAR/RXR	(24, 30)
	Estrogen	Estrogen ERE	(12–14, 31)

Inhibitors	BMP6	SMAD6	(32)
	ZNF521	Promoter repression	(33)
	ZNF423	Autoregulatory	(34)
	miR-195a	3′UTR	(35)
	bta-miR23a	3′UTR	(36)
	Neurofibromin 1	RAS/MEK	(37)
	WISP	WNT bone morphogenic protein (BMP)/SMAD	(38)
	PCR2	H3K27methylation	(39)
	Epigenetic	CpG island methylation	(40)

Co-interacting	Early B cell factor	Transactivation	(17, 41, 42)
	SMAD1/4	BMP	(18)
	RAR/RXR	Retinoids	(24, 30)
	Notch (notch intracellular cytoplasmic domain)	Notch	(43)

Targets	Poly(ADP-ribose) polymerase 1	BMP/SMAD	(44)
	Xvent	BMP/SMAD	(18)
	SMAD6	BMP/SMAD	(32)
	Hes5	Notch	(43)
	TULP3	Sonic Hedgehog	(45)
	Peroxisome proliferator activated receptor-γ	Adipogenesis	(46–48)
	BRCA1	Estrogen	(12–14, 31)

Degradation	CTSO	Estrogen	(13)

In neural development, there is considerable evidence supporting a role for ZNF423 in midline patterning of the central nervous system; consistently, homozygous *zfp423* deletion pups were found to be ataxic and having defects in the cerebellum, forebrain, and olfactory bulb, which could be attributed to a Purkinje cell intrinsic defect (49–53). Mechanistically, sustained Zfp423 expression results in restraint of olfactory neuronal differentiation through the inhibition of EBF activity (41, 42). In *Xenopus*, ZNF423 binds BMP-activated SMAD 1 and 4 proteins, resulting in the activation of the *Xvent-2* promoter to regulate mesoderm and neural development (18). Modulation of the inhibitory SMAD 6 by ZNF423 alters the intensity and duration of the BMP-dependent pathway (32). In the BMP pathway, Poly(ADP-ribose) polymerase 1 (PARP1) acts as a transcriptional coactivator for ZNF423 (44). Zfp423 also has been described to have a role in the Notch pathway, which is important for neurogenesis, by directly interacting with the intracellular cytoplasmic domain of Notch (notch intracellular cytoplasmic domain), thus resulting in upregulation of the Notch target Hes1 (43). In cerebellar granule cell precursors, zfp423 activates sonic hedgehog function *via* the regulation of Tulp3 in the primary cilium (45).

In adipogenesis, Zfp423 is found to be highly expressed in pre-adipocytes compared to non-committed NIH3T3 fibroblasts, and during adipogenesis, the activation of Zfp423 was potentiated by BMP/SMAD signaling and resulted in transactivation of peroxisome proliferator activated receptor-γ (PPARγ), a key transcription factor needed for the maturation of adipocytes (46–48). This function of Zfp423 is controlled by WNT1-inducible signaling pathway protein 2 (WISP2), a WNT pathway protein, which forms a WISP2–Zfp423 complex in the cytoplasm, which can be dissociated with BMP4/SMAD signaling, thus allowing Zfp423 to enter the nucleus for transcriptional activation of target genes (38). Fetal development of subcutaneous white adipose tissue is dependent on Zfp423 (54), through suppression of the beige cell thermogenic program (55). Epigenetic modifications reprogram Zfp423 expression in fetal mouse adipocyte differentiation (56). The ZNF423 expression in the stromal vascular fraction of human pre-adipocytes from non-obese individuals was found to be inversely correlated with the size of the subcutaneous adipose cells, and low ZNF423 expression was associated with adipose cell hypertrophy and with an insulin resistant phenotype (40). Regarding the mechanisms of posttranscriptional regulation, mRNA for Zfp423 was found to be targeted by miR-195a, an anti-adipogenic regulator, which targets and inhibits the 3′UTR (35). Additionally, microRNA profiling in fetal bovine skeletal muscle has recently identified bta-miR23a as an anti-adipogenic regulatory factor in intramuscular adipogenic commitment acting by targeting ZNF423 (36).

On the other hand, the ZNF423 homolog ZNF521 has an inhibitory influence on adipogenesis and inhibits the promoter of *ZNF423* (as discussed below): importantly, ZNF521 downregulation at the onset of adipogenesis is accompanied by ZNF423 mRNA and protein increase, which triggers adipocyte differentiation (33).

## ZNF423 in Human Malignancies

In neuroblastomas (NB), an RNA interference genetic screen identified ZNF423 as critical for retinoic acid-induced differentiation. Specifically, ZNF423 co-activates the RAR/RXR receptor after retinoic acid binding, then promoting differentiation (24, 30). Additionally, the loss of the neurofibromin 1 (NF1) tumor suppressor induces activation of RAS-MEK signaling, which in turn repressed ZNF423 mRNA and protein expression (37). Clinically, retinoic acid is used to treat various cancers, including NB, to overcome their differentiation block. In these studies, low ZNF423 and NF1 expression levels were found to correlate with poor outcome of NB patients. In malignant gliomas, polycomb dysregulation promotes invasion and de-differentiation. In these tumors, the analysis of transcriptional networks identified Zfp423 as a critical factor, whose silencing negatively influenced survival in a SMAD-dependent fashion. Importantly, low ZNF423 expression correlated with poor prognosis in low-grade gliomas (39).

*Zfp423* has been described as an oncogene, and its gene is a frequent retroviral integration site in murine B-cell lymphomas, resulting in ectopic activation and overexpression of Zfp423, which inhibits B cell differentiation though its interaction with EBF (57). When chronic myelogenous leukemic (p210BCR/ABL) mice were crossed with BXH2 mice that transmits a replication-competent retrovirus, an aberrant expression of Zfp423 was observed. Moreover, enforced ZNF423 expression resulted in a cooperative effect with BCL/ABL for the development of chronic myelogoid leukemias with blast crisis (58). In ETV6-Runx1 negative B precursor acute lymphoblastic leukemia (ALL), aberrant expression of ZNF423 induced by epigenetic deregulation inhibits EBF target genes and leads to B cell maturation arrest associated with poor outcome (59). In Epstein–Barr virus infected nasopharyngeal carcinoma, it was found (60) that 8.3% had chimeric fusion rearrangements of Ubiquitin protein ligase E3 Component N-Recognin 5 (UBR5) with the C-terminal domain of ZNF423 (ZF29–30) involved in EBF binding; such chimeric protein increased anchorage-independent growth and tumor formation in nude mice. The oncogenic potential of the UBR5–ZNF423 fusion protein may be related to modulation of EBF activity.

In B-cell leukemias, aberrant ZNF423 induction increases EBF-binding and strongly blocks its action in determining B-cell differentiation, such that leukemia onset is favored and prognosis is poor. In normal hematopoietic progenitors, ZNF521 restrains B-cell differentiation (22, 25, 61–63) and is progressively downregulated to allow differentiation to occur with the correct succession of transcription factors needed.

In nephronophthisis-related ciliopathies, which are degenerative recessive diseases, mutations of either ZNF423 and Centrosomal Protein 164 (CEP164) or meiotic recombination 11 (MRE11) resulted in alteration of DNA damage pathways (64). Mutated forms of ZNF423 were unable to interact with the DNA ds-damage sensor PARP1, and knock-down of *ZNF423* caused an increased sensitivity to DNA damaging agents. In Joubert syndrome with oculorenal anomalies, several mutations for *ZNF423* have been identified including homozygous missense mutation of pP913L and heterozygous truncating mutations, pP506fsX43 and pH1277Y (64). Mutations of the mouse ortholog *Zfp423* cause reduced proliferation and abnormal development of midline neural progenitors resulting in a loss of the cerebellar vermis (50, 51) similar to that seen in Joubert syndrome patients with cerebellar vermis hypoplasia. A mosaic gain of chromosome 16 (16q11.2–16q12.1) involving the *ZNF423* and the *Cerebellin-1* gene was found in a child with intellectual disability, microcephaly, and cerebellar cortical dysplasia (65).

## Transcriptional and Epigenetic Regulation of *ZNF423*

The *ZNF423* gene has been described to have two alternative promoters (21, 59), with the originally studied α-form (18) lacking the NuRD-interacting domain at the N terminus present in the β-form corresponding to the murine Zfp423 transcript (22, 66) and to the homolog *ZNF521/Zfp521*. These forms have differential expression; indeed, it is found that embryonic stem (ES) cell lines express high levels of the β-form, whereas both are present in primary ALL leukemias (67). Both forms are well able to inhibit the transcriptional activation by EBF and are BMP-responsive.

**Figure 1:**
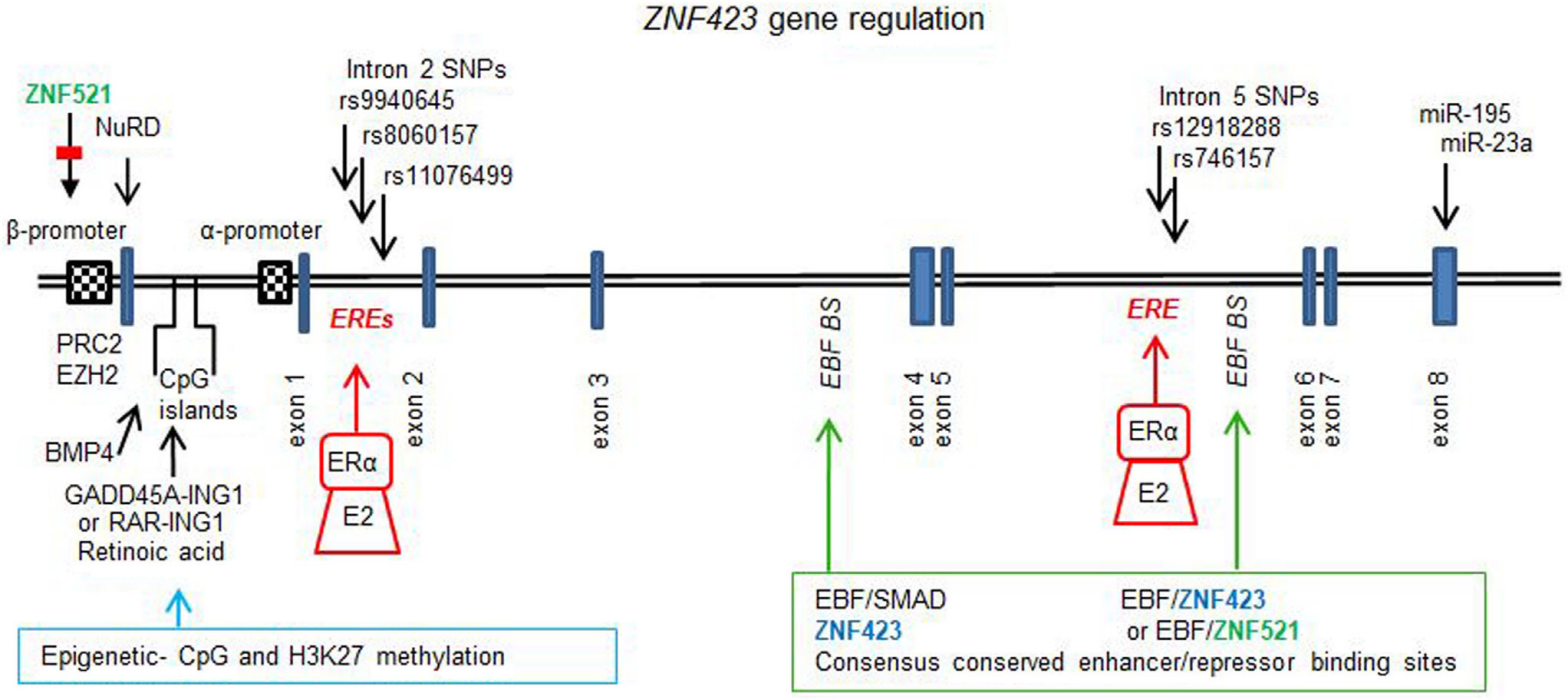
*ZNF423* gene regulation. Diagram to illustrate the structure for the *ZNF423* human (NG_032972.2, 377, 389 bp) gene, which comprises 8 exons and introns with the major part of the protein being coded by exon 4. Two alternative promoters have been described (21, 59), and these are subject to regulation by demethylation and methylation of the CpG islands controlled by GADD45-inhibitor of growth protein 1 (ING1) or retinoic acid induced RAR–ING1 complexes (68). Epigenetic reprogramming is also associated with histone methylation (H3K9me3) and acetylation (H3K9ac), controlled by PCR2 and EHZ2 (56) as well as by ZNF521 (33). BMP4 can result in the demethylation of specific CpG dinucleotides resulting in activation of transcription (40). Consensus conserved across species sites for ZNF423, early B cell factor (EBF), and small mother against decapentaplegic have been found in introns 3 and 5. The enhancer site in intron 5 was found to be occupied by Zfp423 or Zfp521 together with EBF1 resulting in autoregulation by Zfp423 (34) or repression by Zfp521 (33). In ER+ breast cancers, single nucleotide polymorphisms in intron 2 (12) and in intron 5 (15) have been found, which are sufficiently close to ERα canonical consensus-binding sites [estrogen response element (ERE)] such that the response to E_2_ and selective estrogen receptor modulators is affected. Additionally, the 3′UTR of the ZNF423 mRNA contains sites for miR195 (35, 53) and miR23a (36), which control expression.

Figure 1 illustrates the gene for *ZNF423* and the different factors that regulate expression, which are discussed below in-depth. The upstream regulatory region of the *ZNF423* gene locus contains three CpG islands, two of which are found upstream of the β isoform and the third one in between the two *ZNF423* promoters. Both of these promoters are enhanced in conjunction with the CpG islands. Methylation at multiple positions in these CpG islands affects the transcription in ES cells, and ALL leukemias bearing high ZNF423 expression display hypo-methylated CpG islands as compared to normal, lymphopoietic progenitors (67).

Biochemical studies demonstrated that Zfp521 interacted with the mouse *zfp423* promoter (human β promoter) (33). The silencing of *Zfp521* was associated to a loss of H3K9 tri-methylation and an increase in the acetylation of H3K9. The *Zfp423* promoter could be activated by silencing *Zfp521* or by EBF, and this activation is counteracted by overexpression of Zfp521. Knock-down of *Zfp521* in mesenchymal precursor cells resulted in an increased expression of Zfp423, which functionally cooperated with EBF and PPARγ to trigger adipocyte differentiation.

A methylation analysis of the *Zfp423* promoter in obese mice during pregnancy showed a lower H3K27 tri-methylation and higher expression of Zfp423, which predisposes the offspring to obesity, and which was dependent on a reduced binding of enhancer zeste 2 to the *Zfp423* promoter (56). Recently, it has been found that retinoic acid blocks demethylation of the *Zfp423* promoter and induces the partnering between RAR and tumor suppressor [inhibitor of growth protein 1 (ING1)], thus preventing the formation of growth arrest and DNA damage inducible 45 alpha and ING1 complex necessary for locus-specific *Zfp423* DNA demethylation (68, 69). This mechanism has been postulated to contribute to the block in adipogenesis promoted by retinoic acid.

Epigenetic modifications of the *Zfp423* promoter have been characterized in uncommitted NIH3T3 and 3T3-L1 committed pre-adipocytes: in the NIH3T3 cells with very low Zfp423 expression, promoter CpG islands were highly methylated, whereas, they were extensively de-methylated in the 3T3-L1 pre-adipocytes with significant Zfp423 expression (40). When methylation was inhibited by 5-azacytidine treatment, there was a rescue of the differentiation capacity of the NIH3T3, as evident by the induction of Zfp423 and of a number of adipocyte markers. It was also demonstrated that BMP4 could specifically cause the demethylation of the CpG dinucleotide at position −1,016 bp from the transcription start of *Zfp423* promoter, thus resulting in transcriptional activation (40).

A systematic study has been performed to find consensus binding sites for Zfp423, EBF, and SMADs in close proximity in non-coding genomic DNA, based on cross-species conservation of clustered consensus motifs (34). Two of these sites were identified within intron 3 and 5 of the *Zfp423* gene. In intron 3, sites for Zfp423 were close to those for EBF and SMADs and in intron 5 DNA-binding sites for Zfp423 and EBF only were found. These regions were demonstrated to be occupied by Zfp423 itself, and the site in intron 5 was functionally repressed by the overexpression of Zfp423 with EBF, indicating an auto-regulatory feedback loop. Additionally, it has been shown that the homolog Zfp521 could be recruited and represses this site in intron 5 of *Zfp423* (33).

An extensive analysis of the activin-A pathway (70), which is activated in BC, highlighted high inhibin β A (INHBA) and phosphorylation of SMAD2 and 3, whereas other genes involved in the BMP pathway, including ZNF521, were under-expressed. ZNF521 has not been described to be induced by estrogen, whereas ZNF423 can be induced by estrogen and promotes BRCA1 expression, as discussed below.

## SNPs in the Introns of the *ZNF423* and *CTSO* Genes: Relevance in ER+ BC

A genome-wide association study (GWAS) has been undertaken to identify SNPs in BC risk during therapy with SERMs (12). The GWAS was performed from the double-blind placebo-controlled National Surgical Adjuvant Breast and Bowel Project (NSABP) P1 and P2 BC prevention trials, which involved 592 participants who developed BC undergoing SERM therapy with either tamoxifen or raloxifene, and 1,171 matched controls. The two trails showed that tamoxifen and raloxifene reduced BC occurrence by 38–50%; however, preventive SERM therapy has not been widely used, partly due to rare but serious side effects and a large number of women who must be treated to prevent one case of BC (71).

A total of 592,236 SNPs were analyzed (12), with the most significant being from chromosomes 16, 13, and 4. The SNPs on chromosome 13 was not close to any known gene, whereas the SNPs on chromosomes 16 and 4 were either in or near the *ZNF423* gene and the *CTSO* gene encoding *CTSO*. The alleles for rs8060157 and rs11076499 SNPs on Chr 16 mapped to the intron 2 of *ZNF423* and were associated with a decreased risk for BC development with SERM therapy (odds ratio 0.70). The two SNPs identified for *ZNF423* were in addition to 8 SNPs found also intron 2 but with *p*-values of less significance (2.12e−4.22e−06). Conversely, the SNP for *CTSO* on Chr 4 (rs10030044) was associated with an increased risk for developing BC with an odds ratio of 1.42. Although the *p*-values for these SNPs did not reach genome wide significance (i.e., *p* < e−07), they were close to the limit, such that a detailed functional investigation was justified.

Since the SNPs were associated to SERM treatment, experiments were performed to establish a relationship with estrogen treatment and response. In the estrogen-dependent BC cell lines ZR75-30 and ZR75-1, it was found that both ZNF423 and BRCA1 (Breast Cancer 1) could be induced by estradiol (12).

To establish a link with the specific genotypes, a set of lymphoblastoid cell lines (LCLs) (a panel of 300 LCLs with dense SNP and mRNA expression data) were used (12). Some of these cell lines, either WT or with the variant SNPs were stably transfected with ERα to test for E_2_ response. Similar to the BC cell lines, the LCLs were found to induce ZNF423 and BRCA1 under estradiol treatment, and this induction occurred only in cell lines with the WT genotype, whereas when the variant SNPs were present, inductions were only minimally detected.

By using a promoter-reporter construct with the 5′-flanking region of the *BRCA1* promoter, it was established that ZNF423 could induce the transcription of the *BRCA1* gene (72). Indeed, in the promoter region, it was possible to identify four specific consensus sites (CCGCCC) for ZNF423–DNA interaction (17), one of which was bound specifically by ZNF423 after estradiol treatment, as documented by chromatin immunoprecipitation assays.

An *in vitro* model (12) predicting SERM response was developed by using LCLs cell lines treated with increasing concentrations of E_2_ or with 4-OH-TAM (4-hydroxytamoxifen, an active tamoxifen metabolite), a modulator of the estrogen response. LCLs homozygous for WT SNP genotypes exhibited an increase of ZNF423 and BRCA1, whereas ZNF423 and BRCA1 upregulation was reversed to baseline levels upon estrogen receptor blockade. Interestingly, when the specific *ZNF423* SNPs were present, neither ZNF423 nor BRCA1 were induced by E_2_, while mRNA levels were then increased with SERM blocking. This observation was in line with the GWAS study demonstrating the *ZNF423* SNPs having an increased “estrogen” response, generated by the ERα downstream transcription factors ZNF423 and BRCA1.

**Figure 2:**
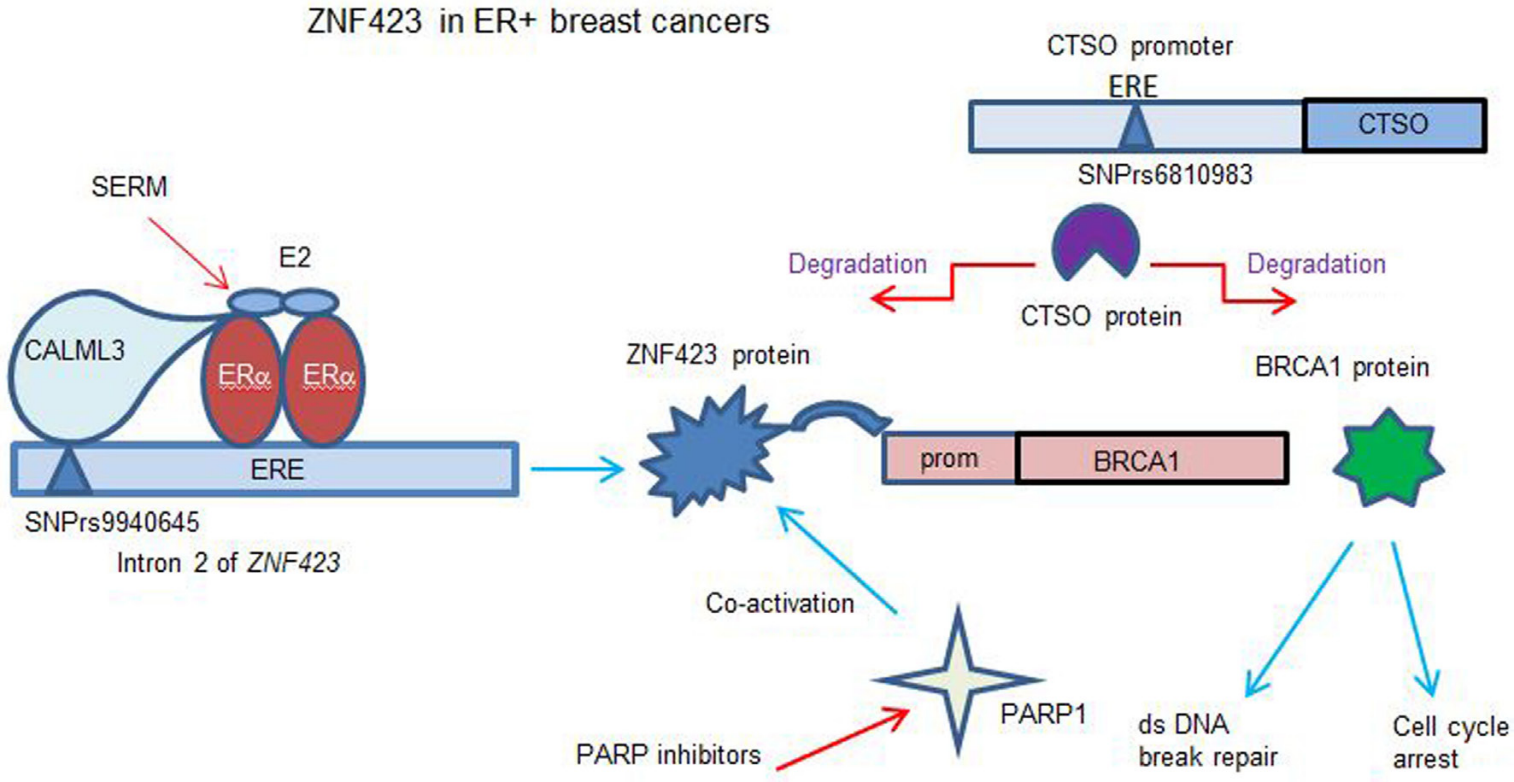
Model for role of *ZNF423* and *CTSO* genes in controlling BRCA1 in ER+ breast cancer (BC). The results (12–15) show that E_2_ can induce ZNF423 transcription critically requiring a region in the *ZNF423* intron 2 near to four canonical, estrogen response element (ERE) sites. The calmodulin like 3 protein was found to act as a sensor to connect the single nucleotide polymorphism (SNP) DNA site with activated ERα. ZNF423 in turn induces the expression of BRCA1 acting through a region of the *BRCA1* promoter, which has four ZNF423-binding sites. This process is modulated in variants that display SNPs in the *ZNF423* intron 2, such that the normal estrogen response is reduced, whereas the response to selective estrogen receptor modulators (SERMs) can now have a reverse effect inducing ZNF423 and then BRCA1. This is compatible with the ZNF423 SNPs being associated with a decreased risk for BC during SERM therapy. The *CTSO* promoter has several SNPs from the genoma-wide association study with an increased risk for BC with SERM therapy (14). CTSO degrades specifically ZNF423 and BRCA1, consequently reducing dsDNA break repair and increasing proliferation. The response to SERMs treatment is ineffective as the variant SNP for CTSO interrupts an ERE site in the CTSO promoter (13). Cells with a deficit or low BRCA1 become being addicted to other DNA repair pathways and were more sensitive to PARP inhibitors. Additionally, ZNF423 has also been shown to co-activate Poly(ADP-ribose) polymerase 1 required for DNA break repair (44).

An analysis of intron 2 in the *ZNF423* gene (12) (Figures 1 and 2) showed four canonical ERE estrogen-response elements binding sites, none of which was directly disrupted by the SNPs. Of these, the rs9940645 SNP lies in the region 240 bp distant from the ERE site. Although this SNP was not associated with decreased risk in GWAS study (rs8060157 and rs11076499), it was among the 8 SNPs with low *p*-values. By chromatin immunoprecipitation assays, this region was found to be pulled down by anti-ERα antibody in the presence of E_2_, and this binding was diminished by SERMs. When the variant SNP was present, the binding of ERα to this site was partly abolished in the presence of E_2_, but increased by SERM. A luciferase reporter assay with 500 bp of the intron 2 region (enhancer) followed by 1,500 bp of the *ZNF423* promoter (Beta) confirmed that the variant SNP affected the differential activation by estrogen alone or in association with the addition of 4-OH TAM or raloxifene.

DNA double-strand break repair is a relevant function of BRCA1 for decreased cancer risk. Specifically, it was found that similarly to BRCA1 silencing, knock-down of ZNF423 or CTSO also resulted in an increased level of DNA breaks. In the absence of ZNF423-induced BRCA1 expression, enforced overexpression of BRCA1 could result in effective dsDNA repair. Instead, when CTSO was knocked down and BRCA1 is overexpressed, the degradation determined by CTSO was minimal and BRCA1 could act on DNA repair (12, 13).

The mechanism has been studied (14) by which the rs9940645 *ZNF423* SNP could act to determine the E_2_-dependent response with the nearest ERE site over 200 bp away. Electrophoretic mobility shift assays with probes for the *ZNF423* SNP site (WT or VV) were used to identify DNA-binding proteins interacting in the presence of E_2_ for the WT or variant SNPs. Among the proteins identified by mass spectroscopy, Calmodulin-like protein 3 (CALML3) was required for the E_2_ response as well as for the reversal of SERM effects (14). CALML3 is a calcium sensing protein highly expressed in breast, prostate, and skin epithelial cells and is downregulated in BCs and transformed cells in culture. CALML3 has been shown to be a regulator of myosin-10 and to have a role in cell adhesion and motility. Paired BC cell lines obtained by CRISPR engineering and with an isogenic background having only the SNP variant genotype, as well as the LCLs lines were used to show (14) that knocking down of CALML3 abolished the SNP-dependent gene regulation of ZNF423 and BRCA1 expression in cells treated with E_2_ alone or with the addition of the SERMs. The effect was limited to ZNF423 and BRCA1, while other canonical targets of ERα such as estrogen-responsive finger protein, cytochrome *c* oxidase subunit 7A-related protein, and estrogen receptor-binding site associated, antigen, 9 did not significantly change after CALML3 knock-down (73). The direct binding of CALML3 to the SNP site did not require calcium whereas the interaction with the ERα was calcium dependent. It was thus proposed that CALML3 acts as sensor at the ZNF423 SNP site in intron 2, and once bound, it co-regulates the transcriptional activity of ERα at the nearby ERE sites (Figure 2).

Breast cancer patients with a deficit for BRCA1 are known to display an increased sensitivity to PARP inhibitors or DNA-damaging agents such as platinum compounds. As BRCA1 is transcriptionally regulated by ZNF423, it was tested whether knock down of ZNF423 or CALML3 could increase sensitivity to chemotherapeutics (14). Analysis of cell viability and colony formation in ZNF423 or CALML3 knocked-down clones showed that the cells were more sensitive to the PARP inhibitors olaparib and cisplatin, consistent with a BRCA1 decrease. *In vitro* and *in vivo* assays using a xenograft model indicated that ZR75-1 BC cells with the variant SNP genotype were more sensitive to the PARP inhibitor treatment alone, whereas the WT genotype were more sensitive to the PARP inhibitors in combination with SERMs. Considering that the minor allele frequency of the *ZNF423* SNP rs9940645 was 39% in Caucasian subjects in the GWAS study, a genotype assessment for this SNP could be useful in the selection of different treatment modalities for BC patients.

Next generation sequencing has been used (15) to further identify sequence variations in the areas surrounding the top two SNPs identified (12) across 500 kb of the *ZNF423* and *CTSO* genes. A nested cases-control cohort was selected from the GWAS samples from the P1 and P2 SERM prevention trials. Among novel SNPs identified, 9 were in the *ZNF423* and 12 in the *CTSO* gene within 500 bp of a ERE motif. In intron 5 of *ZNF423* (Figure 2), two SNPs rs746157 (*p* = 8.44e−04) and rs12918288 (*p* = 3.43e−03) in linkage equilibrium were found to be downstream from an ERE site (distances of 196 and 401 bp). Using the LCLs cells homozygous for WT or variant SNPs, chromatin immunoprecipitation assays for ERα showed a reduction in binding of the VV compared to the WT in the presence of E_2_ as well as the reversed effect upon treatment with E_2_ and SERMs. ZNF423 and BRCA1 were both induced by these SNPs independently from E_2_; however, there was a distinct SERM response in the presence of E_2_ such that both ZNF423 and BRCA1 were induced more with the variant SNPs. This pattern of response is different to that seen with the SNP located upstream from the ERE sites in intron 2 where the variant did not result in an induction in response to E_2_ alone. It is not known whether CALML3 (14) could also mechanistically be involved at these sites in intron 5. It has been known for a while that DNA sequences variation near ERE sites can alter ERα binding (31) but with the novel SNPs described, the distance from the actual ERE site is considerably longer, several hundreds of bases (200–400 bp) compared to directly flanking sequences. Intron 5 has also been implicated in the autoregulation of zfp423 in conjunction with EBF at conserved consensus sites for Zfp423 and EBF (34) as well as by the homolog zfp521 functionally important in adipocytes for control of zfp423 (33) (Figure 2).

An additionally 10 nonsynonymous SNPs (nsSNPs) have been identified by next generation sequencing (15) in the gene for *ZNF423* and one in *CTSO*. One of the *ZNF423* nsSNPs resulted in substitution of amino acid Arg617Gln, which results in an ZNF423 allozyme protein present at a reduced level. This allozyme was not more susceptible to degradation than the WT protein, but was derived from a considerably reduced amount of mRNA for ZNF423 with this nsSNP. However, whether this mutation could specifically affect the activity of ZNF423 which remains to be investigated.

A recent functional study has been performed to understand the mechanism underlying the phenomena associated with the *CTSO* and *ZNF423* SNPs (13). Of the SNPs identified for *CTSO*, one interrupted an ERE site, and another was in relative close proximity. When these variant regions were cloned with the CTSO promoter upstream of a luciferase reporter, a transcriptional activation was observed. Overexpression of CTSO in BC cell lines resulted in a striking decrease in BRCA1 protein as well as ZNF423 protein. This is likely to be due to increased protein degradation mediated by the cysteine protease activity of CTSO, and could be inhibited by the specific cathepsin inhibitor E-64. Knockdown of *CTSO* results in an increased BRCA1 mRNA and protein expression, and this modulation of BRCA1 expression could be mediated by ZNF423 being no longer degraded by CTSO and able trans-activate the *BRCA1* promoter (12, 17).

Mass spectrometry analysis of proteins (13) that coimmunoprecipitate with CTSO has identified four transcription factors: metadherin (MTDH), poly(A) binding protein cytoplasmic 4 like (PABPC4L), lamin A/C (LMNA) and eukaryotic translation elongation factor 1 alpha 1 (EEF1A1); when these factors were knocked-down, it resulted in a net decrease in BRCA1 mRNA expression. When CTSO was overexpressed, these four proteins were all decreased, presumably degraded by CTSO. BRCA1 expression is under complex control, transcriptionally induced by ZNF423 and degraded by CTSO cysteine protease with these interacting transcription factors (MTDH, PABPC4L, LMNA, and EEF1A1). A deficit of BRCA1 is known to play role in cancer development and results in tamoxifen resistance. Deletion or inhibition of CTSO can increase BRCA1 levels with an increased sensitivity to SERMs, resulting in growth arrest. Silencing BRCA1 together with CTSO reversed this effect abrogating the decreased proliferation. These results suggest that CTSO may regulate cell proliferation and tamoxifen response through BRCA1 (Figure 2).

The GWAS-identified SNPs in the *ZNF423* gene, which were associated with decreased risk and SNPs for *CTSO* with increased risk in SERM-treated BC patients. LCLs (13) carrying the favorable combination (*CTSO* W/*ZNF423* V) were less prone to proliferation, while those carrying the unfavorable (*CTSO* V/*ZNF423* W) had a proliferative advantage. In the presence of only one favorable SNP, the proliferation rate was instead tamoxifen-dependent. Taken together, the findings suggest that patients with only one favorable allele would benefit from tamoxifen therapy. Interestingly, for the tamoxifen-non responsive unfavorable *CTSO* V/*ZNF423*W genotype, the PARP inhibitor treatment restored tamoxifen sensitivity. Identifying the signature of the *CTSO* and *ZNF423* SNPs may, therefore, potentially provide a valuable opportunity for the stratification of ER+ BC types into different drug response subgroups.

A considerable wealth of information has been elegantly derived from genotype specific cell lines, gaining insights into the mechanisms involving the estrogen response for ZNF423 through BRCA1 induction and CTSO degradation (Figure 2). Unfortunately, as yet, these findings could not be translated into clearly establishing the SNPs as biomarkers for SERM therapy prediction. Studies have been performed based on the GWAS data for identification of SNPs with relevance for SERM therapy, by following up a patient panel of 586 BCs in Japan, to establish whether these SNPs could realistically be considered prognostic markers for an increased recurrence rate for patients receiving adjuvant tamoxifen therapy during long term follow-up (74). Kaplan–Meier survival curves showed a significant difference only for the *CTSO* SNP rs10030044 for TT to GG as a poor prognostic factor for disease free or overall survival (*p* = 0.055, *p* = 0.017) in BC positive for hormone receptors. No association of the *CTSO* genotype and the mRNA expression of CTSO and BRCA1 could be established. In this study, the *ZNF423* rs8060157 genotype was not associated with survival or prognosis. Additionally, two randomized prevention trials for BC patients treated with SERM therapy were analyzed for *ZNF423* and *CTSO* SNPs significance (75). The two SNPs for *ZNF423* rs8060157 and rs10030044 for *CTSO* were analyzed together with additional 80 SNPs in the surrounding regions. The IBIS-1 and Marsden trials in the UK involved 369 cases and 662 controls, with 148 cases and 268 controls in the tamoxifen arm; however, the results did not support the predicted response found initially (12), presenting no statistically significant evidence to suggest that such SNPs might predict response to SERMs.

## Conclusion

To have available a medicine, which would prevent or reduce the probability of BC, is an extremely important goal; however, such a therapy, which is given to healthy women, needs to be practically without side effects, such that there is a distinct favorable benefit to risk ratio. The use of SERMs is promising and the statistics from several trails (3–5) indicate a significant reduction in occurrence of BC but concerns over safety remain such that widespread use has not been adopted. The stratification of women according to BC risk, should identify a restricted high risk group, which where the benefit would out weight the risk. The use of sets of SNPs (7, 8, 76), which are currently being identified and validated, as a polygenic risk score as part of the risk classification will aid this stratification. The sets of these SNPs are now considered to account for just 18% of the heritability of BC.

Additional biomarkers that can reliably predict a response to a preventive therapy will be required to obtain information that better defines the ideal group that will benefit from treatment. In this context, it is paramount that the molecular mechanisms in act to obtain the SERM response and its reversal are well understood. The papers described in this review (12–15) delineate a role for the transcription factor ZNF423, together with CALML3 to effectuate and control the response to estrogens and SERMs. This process is dependent on intronic sequences in the *ZNF423* gene near to ERE sites. Estrogen was found to induce the expression of ZNF423, which could in turn transactivate the promoter of *BRCA1* increasing its expression and activity. It can also be predicted that epigenetic regulation, such as methylation of CpG islands and methylation/acetylation of histones resulting in nucleosome rearrangements at the *ZNF423* gene (40, 55, 56), will also influence the ZNF423-dependent estrogen response in hormone-responsive BC.

This role for ZNF423 in the mechanism of action of estrogen was only explored after the SNPs in the intron of ZNF423 were found to have low *p* values in the BC prevention trails NSABP P1 and P2 in the USA (12). A polygenic risk score was obtained from this trial (12, 76) where 75 SNPs were analyzed and it was tested (76) whether the effect of additionally including the rs10030044 in *CTSO* and rs8060157 in *ZNF423*; however, these did not modify the score obtained. The fact that these SNPs did not emerge statistically in trials in the UK and in Japan could be due to ethnic bias, which has been noted for certain SNPs between divergent populations (10). The strategy has since been to analyze sets of SNPs as a polygenic analysis, these groups where each individual SNPs has a relatively small preventive prediction factor together created a statistic value with greater weight. However, these groups of SNPs contain some without any known association with estrogen response genes and should inspire studies that may reveal the underlying mechanism. Frustratingly, where a molecular justification for the importance of the specific SNPs for ZNF423 and CTSO has been elucidated, in fact, the statistical correlation for prevention was insufficient for inclusion in the polygenic SNP set analysis.

The increasing identification of nucleotide variations in GWAS in evergrowing numbers of patients and controls, which are then followed for a considerable number of years until cancer may develop (10 years data are available for some trials), means that it might be anticipated that predicative values from SNPs with a molecular basis for modulating the response to SERMs will emerge as more robust factors. Considering the induction of ZNF423 in ER+ BC with estrogen and its ability to induce BRCA1, future studies may concern an evaluation of the expression in specific types of BC as well as in ovarian cancer and if ZNF423 could be considered a useful biomarker or a therapeutic target in conjunction with SERMs or with PARP1 inhibitors.

## Author Contributions

HB wrote the draft of the manuscript. SS, EC, NA, VL, AA, and YM read the literature and discussed the findings. NA, MM, and GM revised the manuscript.

## Conflict of Interest Statement

The authors declare that the research was conducted in the absence of any commercial or financial relationships that could be construed as a potential conflict of interest.

## Abbreviations

ALL, acute lymphoblastic leukemia; BC, breast cancer; BCL11/Evi9, B-cell CLL/lymphoma 11 A; BCR/ABL, breakpoint cluster region/abelson proto-oncogene; BRCA1, breast cancer 1; BMP, bone morphogenic protein; BRE, BMP responsive element; CALML3, calmodulin like 3; CBLN1, cerebellin-1; CEP164, centrosomal protein 164; COX7RP, cytochrome *c* oxidase subunit 7A-related protein; CTSO, cathepsin O; EBAG9, estrogen receptor binding site associated, antigen 9; EBF, early B cell factor; EEF1A1, eukaryotic translation elongation factor 1 alpha 1; EFP, estrogen-responsive finger protein; ER, estrogen receptor; ERE, estrogen response element; ES, embryonic stem cells; ETV6-Runx1, Ets variant gene 6 (TEL oncogene)/runt related transcription factor 1; FOG1, friend of GATA1; GADD45A, growth arrest and DNA damage inducible 45 Alpha; GWAS, genoma-wide association study; INHBA, inhibin β A; ING1, inhibitor of growth protein 1; LCL, lymphoblastoid cell line; LMNA, lamin A/C; MRE11, meiotic recombination 11; MTDH, metadherin; NF1, neurofibromin 1; NB, neuroblastoma; NICD, notch intracellular cytoplasmic domain; NuRD, nucleosome remodeling domain; NSABP, national surgical adjuvant breast and bowel project; OAZ/ROAZ, rat Olf/EBF-associated zinc finger protein; PABPC4L, Poly(A) binding protein cytoplasmic 4 Like; PARP1, Poly(ADP-ribose) polymerase 1; PPAR γ, Peroxisome proliferator activated receptor-γ; RAR/RXR, retinoic acid receptor/retinoid X receptor; RB, retinoblastoma; RBBP7/4, RB-binding protein 7/4; Sall1, spalt like transcription factor 1; SERM, selective estrogen receptor modulator; SMAD, small mother against decapentaplegic; SNP, single nucleotide polymorphism; UBR5, ubiquitin protein ligase E3 component N-recognin 5; WISP2, WNT1 inducible signaling pathway protein 2; WNT1, wingless-type MMTV integration site family, member 1; Zfp, zinc finger protein.; ZNF, zinc finger.
